# Early Warning Signs, Effects, Risk Factors, and Diagnostic Indicators of Toxoplasmosis in Pregnant Women in Africa: A Scoping Review

**DOI:** 10.3390/tropicalmed11040104

**Published:** 2026-04-17

**Authors:** Cherotich Jesca Tangus, Ndichu Maingi, James Chege Nganga, Davis Karanja Njuguna, Kariuki Njaanake, Bruno Enagnon Lokonon, Gloria Ivy Mensah, Kennedy Kwasi Addo, Andrée Prisca Ndjoug Ndour, Bassirou Bonfoh

**Affiliations:** 1 Department of Veterinary Pathology, Microbiology and Parasitology, Faculty of Veterinary Medicine, University of Nairobi, Nairobi P.O. Box 29053-00625, Kenya; 2 Department of Medical Microbiology, University of Nairobi, Nairobi P.O. Box 19676-00202, Kenya; 3 Laboratoire de Biomathématiques et d’Estimations Forestières (LABEF), Faculty of Agronomic Sciences (FSA), University of Abomey-Calavi, Abomey-Calavi P.O. Box 04 BP 1525, Benin; 4 Centre Suisse de Recherches Scientifiques en Côte d’Ivoire (CSRS), Abidjan P.O. Box 01 BP 1303, Côte d’Ivoire; 5 Noguchi Memorial Institute for Medical Research, University of Ghana, Accra P.O. Box LG581, Ghana; 6 École Inter-États des Sciences et Médecine Vétérinaires (EISMV), Dakar BP 5077, Senegal

**Keywords:** toxoplasmosis, maternal signs, pregnancy & foetal outcomes, risk factors, diagnosis, Africa, scoping review

## Abstract

Toxoplasmosis is a widely distributed zoonosis caused by the protozoan parasite *Toxoplasma gondii.* Infection during pregnancy is a major public health concern due to its potential impact on both maternal health and fetal development. Early detection of maternal infection is critical to prevent adverse outcomes; however, maternal signs are often subtle, non-specific or absent, complicating timely diagnosis. This scoping review aimed to map and synthesise existing evidence on early maternal signs, pregnancy and foetal outcomes, frequently assessed risk factors, and diagnostic approaches of toxoplasmosis in expectant mothers in Africa. The review was done in accordance with the PRISMA-ScR guidelines. A literature search of PubMed, Scopus, ResearchGate, and Google Scholar was performed to identify studies published between 2000 and 2025. Retrieved records were managed using Zotero (version 8.0.4) for deduplication and screening. Only English-language studies conducted in Africa and reporting relevant maternal or clinical data were included. A total of 28 cross-sectional studies were included. Lymphadenopathy (25.0%) was the most frequently reported maternal early sign, followed by flu-like illness, asymptomatic infection, low-grade or mild fever, and fatigue or malaise (each 10.7%). Congenital anomalies (50.0%) and miscarriage or spontaneous abortion (42.9%) were the most commonly reported foetal and pregnancy outcomes. Frequently reported risk factors were exposure to cat faeces (57.1%) and ingestion of undercooked or raw meat (42.9%). Diagnostic approaches were commonly enzyme-based immunoassays (78.6%), with limited use of RDTs and molecular methods. These findings suggest the need for improved early detection and prevention strategies in high-risk, low-resource African settings. Enhancing routine screening, health education, and access to appropriate diagnostics are considered. Future studies should consider adopting standardised reporting and integrating sensitive, affordable, rapid diagnostic approaches to enhance early detection and reduce the burden of congenital toxoplasmosis.

## 1. Introduction

Toxoplasmosis, a globally common zoonotic infection, is caused by *Toxoplasma gondii*. It presents a significant health risk to pregnant women and their developing foetuses. A greater disease burden has been reported in many low- and middle-income regions, especially in parts of Africa [1]. Human infection mainly occurs through consuming raw or undercooked meat containing tissue cysts or through accidental ingestion of oocysts shed by infected felids [2]. Although the infection is often asymptomatic or shows mild, non-specific symptoms in the mother, congenital transmission may lead to severe foetal outcomes, including miscarriage, stillbirth, neurological deficits, and ocular abnormalities [3]. Despite its considerable public health impact, toxoplasmosis remains largely neglected in developing countries in Africa, where limited awareness and diagnostic capacity impede timely detection and management of maternal infections [4].

Early recognition of maternal infection is important for prompt diagnosis and timely management to prevent adverse pregnancy and foetal outcomes [5]. Clinical manifestations are often subtle, and exposure risks are diverse, including dietary habits, environmental factors, and socio-demographic factors [6]. Accurate and timely diagnosis relies on serological and molecular methods, yet variation in test availability and sensitivity poses challenges for effective surveillance and clinical care [7].

Regional variations in *Toxoplasma gondii* strains, transmission dynamics, and health system capacity highlight the need for context-specific evidence in Africa. Therefore, this review sought to map and synthesise the existing data on initial maternal clinical manifestation, pregnancy and foetal outcomes, commonly reported determinants, and detection approaches for *T. gondii* infection in expectant women in Africa. By focusing on region-specific evidence, the review identifies knowledge gaps to inform future research, strengthen clinical awareness, and guide targeted public health interventions to reduce the burden of congenital toxoplasmosis in Africa.

## 2. Methodology

This scoping study was conducted in accordance with the PRISMA-ScR guidelines, and a PRISMA-ScR checklist was generated (Table S1) [8]. The review aimed to map and synthesise the existing literature on maternal early warning signs, pregnancy and foetal outcomes, frequently assessed determinants, and diagnostic approaches of *T. gondii* infection in expectant women in Africa. A scoping design was considered appropriate to capture the breadth of existing literature, identify key concepts, and highlight gaps in evidence. The review protocol was developed a priori and is available on the Open Science Framework (10.17605/OSF.IO/PDZBC).

### 2.1. Search Strategy

A thorough literature search was conducted across PubMed, Scopus, Google Scholar, and ResearchGate to identify studies published between January 2000 and 8 December 2025. The initial search was conducted on 7 October 2025 and updated on 8 December 2025. Database-specific search strategies incorporated combinations of Medical Subject Headings (MeSH) and free-text terms, with Boolean operators applied to optimise retrieval. Full, reproducible search strings for PubMed and Scopus are provided in Supplementary File S1 and uploaded to the OSF repository.

Due to platform limitations, Google Scholar searches were conducted using simplified terms, and the first 200 results were screened for relevance. ResearchGate was used as a supplementary source to identify grey literature and retrieve full-text articles not readily accessible through primary databases. Additional manual searches were performed to ensure completeness.

All records were transferred to Zotero for organisation and deduplication using automated and manual verification procedures.

### 2.2. Study Selection and Eligibility

Screening was conducted in two stages: title and abstract screening, followed by full-text review. Studies were included if they: (i) involved pregnant women; (ii) were conducted in African countries; (iii) reported maternal clinical features, pregnancy or foetal outcomes, risk factors, or diagnostic approaches related to toxoplasmosis; and (iv) provided primary data from observational or experimental study designs.

Studies were excluded if they focused solely on non-pregnant populations or animals, reported only seroprevalence without clinical or outcome data, lacked sufficient methodological detail, or were non-English and not published in Africa within the specified timeframe. Reviews, editorials, and inaccessible full texts were also excluded. Studies reporting only foetal outcomes were retained where they contributed to the secondary objective of characterising pregnancy and foetal outcomes.

**Figure 1:**
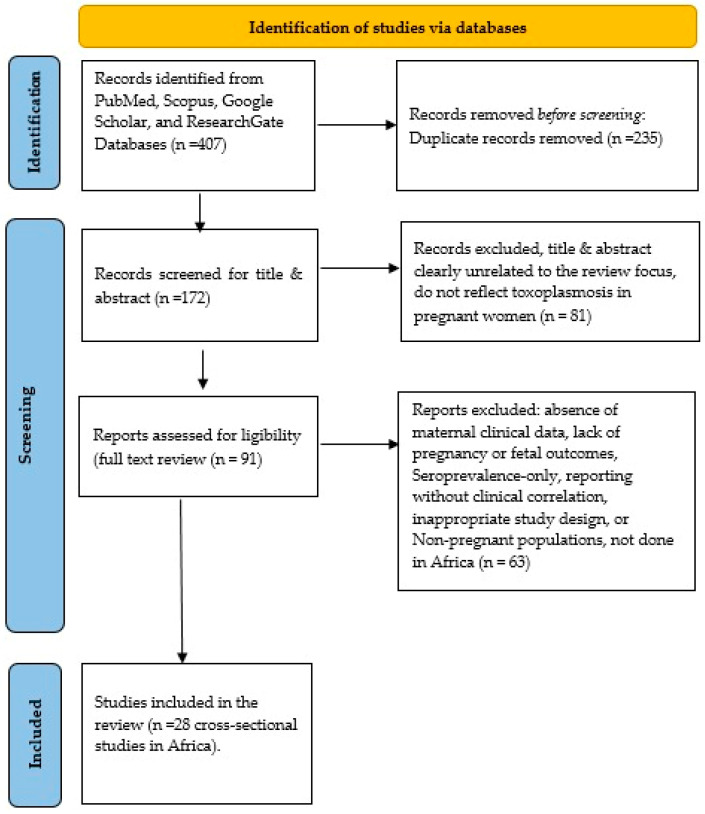
PRISMA 2020 Flow Diagrams modified from: [9].

The study selection process was managed using Zotero and is summarised in the PRISMA flow diagram (Figure 1).

### 2.3. Extraction of Data

Data were extracted using a predesigned and pilot-tested form developed in Microsoft Word 2019, guided by a standardised codebook to ensure consistency and transparency. Extracted information included study characteristics (author, year, location, region, and study design), potential symptoms and effects, reported risk factors, and diagnostic approaches.

Following extraction, the data were transferred to Microsoft Excel for organisation, coding, and verification. All variables were predefined and operationally defined in accordance with the codebook (Supplementary File S1) and the OSF-registered protocol. Where multiple outcomes or diagnostic methods were reported, all relevant data were captured. The dataset was subsequently reviewed for accuracy and completeness before analysis.

### 2.4. Assessment of Risk of Bias

This review aimed to map and summarise the existing data on initial maternal symptoms, frequently reported determinants, and diagnostic methods for *T. gondii* infection in pregnant women in Africa. Given the broad scope of the review and variability in study designs and reporting, a formal risk-of-bias or methodological quality assessment was not conducted. The purpose of this review was to identify patterns, themes, and literature knowledge gaps, but not to produce pooled effect estimates or graded evidence recommendations.

### 2.5. Statistical Analysis

Due to substantial heterogeneity in study design, populations, diagnostic methods, outcome definitions, and reporting formats, quantitative meta-analysis was not undertaken. Comparable effect estimates were inconsistently reported, precluding statistical pooling and formal assessment of heterogeneity.

In line with a scoping review approach, a descriptive and exploratory synthesis was conducted to map the nature and distribution of available evidence. Findings were organised into four thematic categories: (1) maternal early warning signs and symptoms, (2) pregnancy and foetal outcomes, (3) commonly reported risk factors, and (4) diagnostic approaches.

Data were managed in Microsoft Excel and summarised using descriptive statistics. Results are presented as the proportion of included studies reporting each variable, illustrated through tables and figures. This approach enables the identification of key patterns and research gaps rather than estimating causal effects.

### 2.6. Ethical Considerations

This review did not require ethical approval; only open accessible studies were used.

## 3. Results

A total of 407 records were identified from PubMed, Scopus, Google Scholar, and ResearchGate (Figure 1). After removal of 235 duplicates, 172 records underwent title and abstract screening, of which 81 were excluded.

Ninety-one full-text articles were assessed for eligibility, and 63 were excluded due to irrelevance to pregnant women, absence of required outcomes, non-African settings, or insufficient data. The full list of excluded studies with reasons is available in the OSF repository.

Following PRISMA-guided screening, 28 studies were included in the final synthesis (Figure 1). Although multiple study designs were eligible, only cross-sectional studies met the inclusion criteria due to comparability of outcomes and availability of extractable data. These studies provided evidence on maternal clinical features, pregnancy and foetal outcomes, risk factors, and diagnostic approaches to toxoplasmosis in pregnant women in Africa.

The 28 studies included in this review were conducted across African countries. Nigeria (5) contributed the most studies, followed by Ethiopia (4) and Cameroon (3), with other countries represented by one or two studies (Table 1). By region, studies were predominantly from Eastern (9), followed by Western (7), Central (4), Northern (3), and Southern Africa (2).

### 3.1. Maternal Early Signs/Symptoms (Maternal Clinical Manifestations)

**Figure 2:**
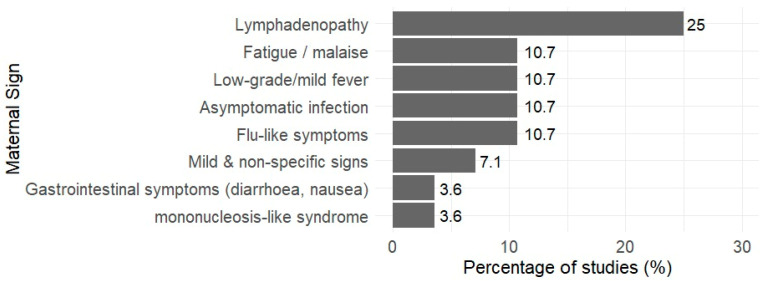
The percentage of the 28 included studies reporting early maternal signs due to Toxoplasmosis (*n* = 28).

Early maternal signs and symptoms were variably and inconsistently reported across the included studies (Figure 2). Lymphadenopathy (swollen glands) was the most frequently documented sign (25.0%). This was followed by flu-like illness, asymptomatic infection, low-grade or mild fever, and fatigue or malaise, each reported in 10.7% of studies. Mild non-specific symptoms were reported in 7.1% of studies, while mononucleosis-like syndrome and gastrointestinal symptoms were least frequently reported, each occurring in 3.6% of studies.

### 3.2. Pregnancy and Foetal Outcomes of Maternal Infection

**Figure 3:**
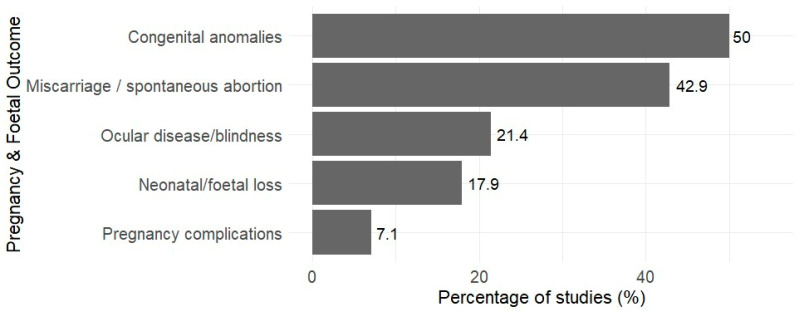
The percentage of the 28 included studies reporting pregnancy and foetal outcomes (*n* = 28).

Adverse pregnancy and foetal outcomes were reported in the included studies (Figure 3). Congenital anomalies, including congenital malformations, hydrocephalus, cerebral calcification, and neurological impairments, were the most frequently reported foetal outcomes (50%), followed by Miscarriage or spontaneous abortion (42.9%). Other reported outcomes included ocular disease or blindness (21.4%) and neonatal or foetal loss (17.9%). Less commonly, pregnancy-related complications such as eclampsia, anaemia, and gestational diabetes were reported in 7.1% of studies.

### 3.3. Commonly Reported Factors for Toxoplasmosis in Pregnancy

**Figure 4:**
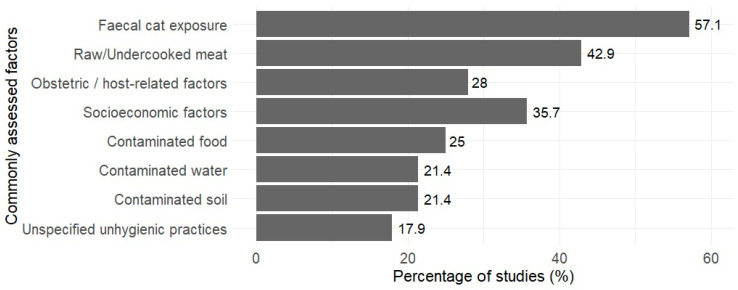
Percentage of frequently assessed factors across 28 studies included in the review (*n* = 28).

Risk factor analysis revealed consistent environmental and foodborne exposures across studies (Figure 4). Frequently assessed risk factors were multifactorial, spanning dietary, environmental, socioeconomic, and host determinants. Exposure to cat faeces was the most frequently reported factor (57.1%), followed by consumption of undercooked or raw meat (42.9%), socioeconomic factors (35.7%), and obstetric and host-related factors (28.0%). Environmental and behavioural factors, including contaminated food (25.0%), contaminated soil and water (21.4% each), and unspecified unhygienic practices (17.9%) were also reported.

### 3.4. Diagnostic Indicators and Methods for Toxoplasmosis

**Figure 5:**
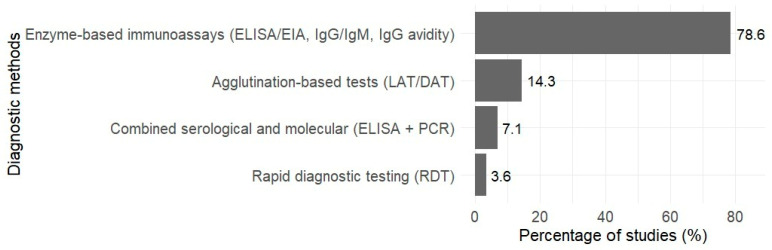
Diagnostic techniques utilised across reviewed studies (*n* = 28).

The included studies reported use of serological methods for diagnosis, with limited use of RDTs and molecular techniques (Figure 5). Enzyme-based immunoassays (ELISA/EIA, IgG/IgM assays, and IgG avidity testing) (78.6%) were the most frequently used diagnostic methods, followed by agglutination-based tests (LAT/DAT) (14.3%), combined serological and molecular approaches (ELISA + PCR) (7.1%). And rapid diagnostic testing (OnSite Toxo IgG/IgM Combo Rapid Test) (3.6%).

## 4. Discussion

Toxoplasmosis is a significant public health concern during pregnancy due to its impact on maternal and foetal health [1]. If maternal infection during pregnancy is not identified and managed promptly, the parasite may cross the placenta and infect the foetus, leading to congenital toxoplasmosis with potentially severe adverse outcomes [1]. This scoping review summarises the reported maternal clinical features, pregnancy and foetal outcomes, commonly identified risk factors, and diagnostic approaches. It also highlights key knowledge gaps and provides suggestions for future research to improve early detection and prevention of congenital toxoplasmosis in high-risk, low-resource settings in Africa.

Maternal early warning signs of toxoplasmosis during pregnancy were generally mild, non-specific, or absent across the included studies. Lymphadenopathy was the most commonly reported sign, followed by flu-like symptoms, low-grade fever, and fatigue, while some cases were asymptomatic. Such presentations are easily overlooked or misattributed to other common illnesses, limiting the reliability of clinical diagnosis alone [11]. This lack of distinct clinical indicators complicates early detection without laboratory confirmation and increases the risk of undiagnosed maternal infection and subsequent vertical transmission, potentially resulting in congenital toxoplasmosis [18].

The most often reported pregnancy and fetal outcomes in the examined studies were congenital abnormalities and miscarriages. Acute toxoplasmosis during pregnancy causes miscarriage and abortion, whereas congenital abnormalities are characterised by foetal problems such as hydrocephalus, chorioretinitis, or cerebral calcifications [26]. This does not, however, imply that they are the most common effects of toxoplasmosis. Their severity increases the possibility of reporting and clinical evaluation. In several studies, they were reported alongside maternal symptoms, though they are secondary findings, not early warning signs, as they represent consequences of vertical transmission rather than early indicators of maternal infection. These findings might reflect the clinical significance of undiagnosed maternal infection and suggest the potential severity of congenital toxoplasmosis. Importantly, toxoplasmosis represents only one of several potential causes of these adverse outcomes [18]. From a scoping perspective, prompt detection of toxoplasmosis in expectant women is therefore important to prevent adverse outcomes.

Exposure to cat faeces was the most frequently reported risk factor, reflecting its established role in environmental contamination and transmission. This emphasises how crucial it is to follow preventative measures like managing litter safely, washing hands frequently, and staying away from stray cats while pregnant [15]. Consumption of undercooked or raw meat was also commonly reported, showing the transmission of the parasite via consumption of the infective stage in muscle tissues [27]. Reported socioeconomic factors reflect conditions in many African settings where low health awareness increases exposure to *Toxoplasma gondii* [4]. This may contribute to unsafe food handling practices and reduce the adoption of preventive behaviours. Obstetric and host-related factors were also reported, suggesting that biological and reproductive characteristics of a pregnant woman may influence susceptibility or detection patterns of toxoplasmosis [22]. Contaminated soil, food, and water were also assessed, further emphasising environmental exposure routes [6]. This highlights the need for integrated interventions, including improvements in hygienic practices, enhancing clean water access, and targeted community health education in African high-risk areas.

The variation in reporting frequency of these factors may reflect differences in questionnaire design, inconsistent exposure definitions, cultural perceptions of risk, and recall bias. As most included studies were cross-sectional, the findings reflect the frequency with which exposures were reported, rather than confirmed causal relationships. All included studies were conducted in Africa, where regional differences in dietary habits, farming practices, human–animal interactions, sanitation standards, and awareness of toxoplasmosis may influence both exposure patterns and reporting practices [21]. Therefore, when extrapolating these results to other geographical areas with distinct epidemiological and sociocultural circumstances, care should be taken.

Given the scoping nature of this review and the absence of formal quality grading, the findings should be interpreted as a descriptive mapping of the available evidence rather than high-certainty conclusions about risk magnitude.

Nevertheless, the consistent identification of preventable exposure pathways supports the need for strengthened public health education targeting pregnant women in African hotspot areas. Integrating awareness campaigns with antenatal screening may promote practical preventive measures among pregnant women, including thorough cooking of meat, proper hand hygiene, safe washing of vegetables, use of clean water, and avoidance of direct contact with cat faeces during pregnancy [11]. Such interventions may be particularly important for pregnant women in many African high-risk settings with limited sanitation infrastructure and diagnostic capacity, which may increase vulnerability to *T. gondii* infection and delay timely detection [24].

Diagnostic approaches were predominantly serological, particularly ELISA/EIA for IgG and IgM detection and, less commonly, IgG avidity testing to distinguish recent from past infection. However, these methods are not routinely available or affordable in many primary antenatal care settings and are more often accessible in better-equipped laboratories, limiting their use for antenatal screening in resource-constrained contexts [18]. Only a limited number of studies employed agglutination-based tests and PCR in combination with ELISA and rapid diagnostic testing. The limited use of molecular diagnostics may be due to costs, infrastructure needs, and the need for specialised laboratory expertise. While serological methods remain valuable for screening, molecular techniques offer direct evidence of parasite DNA and enhance diagnostic specificity, especially in suspected acute or congenital infections [7]. From a scoping perspective, the findings highlight reliance on traditional serology and indicate a need for broader adoption of confirmatory molecular tools to improve diagnostic accuracy in pregnancy-related toxoplasmosis. Therefore, to enhance early warning systems and reduce the burden of congenital toxoplasmosis, future research should integrate enzyme-based immunoassays, RDTs and molecular diagnostics, while strengthening preventative education initiatives in African resource-limited and high-risk areas.

## 5. Conclusions and Recommendation

Strengthening prevention and early detection of toxoplasmosis in pregnancy is considered in African resource-limited settings. Practical strategies include integrating toxoplasmosis education into routine antenatal care, promoting safe food handling and hygiene practices, and training nurses, midwives, and community health workers to deliver targeted risk-reduction messages and basic screening.

Improving access to diagnostic services through centralised reference laboratories, strengthened referral systems, and the phased introduction of appropriate serological tests, rapid diagnostics, and molecular methods within public health laboratory networks may further enhance timely and accurate diagnosis. Future studies should consider adopting clearer and standardised reporting of findings, with distinct classification of maternal signs, pregnancy outcomes, and risk factors, while incorporating more sensitive and affordable diagnostic methods.

## 6. Limitation of This Review

This review has several limitations. As a scoping review, no formal risk-of-bias assessment was conducted, and most included studies were cross-sectional, limiting causal inference and increasing susceptibility to selection, recall, and confounding bias.

There was substantial heterogeneity in study design, exposure definitions, outcome measures, and diagnostic methods. Diagnostic approaches ranged from enzyme-based immunoassays to agglutination-based tests, RDTs and PCR, which may have led to misclassification of acute and chronic infections. Key outcomes, including congenital anomalies and pregnancy complications, were not consistently defined across studies, limiting comparability.

Inconsistent reporting of maternal clinical features prevented pooling of patient-level data. Consequently, findings reflect the proportion of studies reporting common factors rather than pooled prevalence or effect sizes. Due to this heterogeneity, meta-analysis was not feasible, and results are based on narrative synthesis.

Language bias may have been introduced because the review was limited to literature written in English. All included studies were conducted in Africa, and regional differences in parasite strains, transmission patterns, and healthcare systems may limit generalisability to other settings.

Although comprehensive search strategies were used and are provided in the Supplementary Materials, part of the search involved manual identification of studies, which may introduce minor inconsistencies in source attribution.

Despite these limitations, the review provides a structured overview of the existing evidence and identifies important gaps for future research.

## Figures and Tables

**Table 1:** Summary of included studies (*n* = 28).

Authors and Year of Publication	Study Location	Geographical Region	Study Design	Potential Symptoms and Effects	Reported Risk Factors
Sebaa et al. [2]	Algeria	Northern Africa	Cross-sectional	Miscarriages, birth anomalies.	Eat undercooked meat, contact with cats, and previous spontaneous abortions.
Dambrun et al. [10]	Benin	Western Africa	Cross-sectional	Blindness, nervous disorders, abortion.	Host immunity, *T. gondii* strain.
Nguemaïm et al. [11]	Cameroon	Central Africa	Cross-sectional	Malaise, low-grade fever, and lymphadenopathy.	Pet ownership, including handling of their faeces.
Mabeku et al. [12]	Cameroon	Central Africa	Cross-sectional	Mild fever, lymphadenopathy, fatigue.	Low educational level, soil contact, and consumption of raw or unwashed vegetables.
Yamssi et al. [13]	Cameroon	Central Africa	Cross-sectional	Malaise, low-grade fever, and lymphadenopathy.	Pet ownership and handling of cat faeces.
Doudou et al. [14]	Congo	Central Africa	Cross-sectional	Malformations, spontaneous abortion.	Soil contact, cat ownership, consumption of raw meat, and poor hygiene practices.
Mulugeta et al. [1]	Ethiopia	Eastern Africa	Cross-sectional	Mild symptoms.	Contact with cat faeces, consumption of undercooked meat, raw vegetables, and blood transfusion.
Wodage et al. [15]	Ethiopia	Eastern Africa	Cross-sectional	Abortion, stillbirth.	Cat ownership, soil exposure, and lack of awareness of toxoplasmosis.
Jula et al. [16]	Ethiopia	Eastern Africa	Cross-sectional	Miscarriage cerebral calcification.	Drinking unsafe water.
Agma et al. [17]	Ethiopia	Eastern Africa	Cross-sectional	Flu-like illness, abortion, stillbirth, and severe neurological disorders.	Age, educational status, and cat ownership.
Kamal et al. [18]	Egypt	North Africa	Cross-sectional	Foetal loss, neonatal death, and neurological complications.	Socioeconomic, environmental, and dietary factors.
Addo et al. [19]	Ghana	Western Africa	Cross-sectional	Seizures, jaundice, loss of vision, and developmental delay.	Education level, contact with cats, soil exposure, and consumption of raw fruits and vegetables.
Assoah et al. [20]	Ghana	Western Africa	Cross-sectional	Miscarriage.	Level of education, residence, backyard animal farming, unhygienic practices, water sources, and water quality.
Atif et al. [21]	Morocco	Northern Africa	Cross-sectional	Miscarriage and congenital malformation.	Contact with cats and low awareness.
Zakari et al. [22]	Nigeria	Western Africa	Cross-sectional	Blindness and neurological impairment.	HIV-positive status, older age, and urban residence.
Bello et al. [23]	Nigeria	Western Africa	Cross-sectional	Cold and flu, cervical lymphadenopathy and mononucleosis-like syndrome.	Contact with cats and their litter.
Dawet et al. [4]	Nigeria	Western Africa	Cross-sectional	Asymptomatic.	Consumption of undercooked meat, unpasteurized milk, and exposure to cat faeces.
Akpan et al. [24]	Nigeria	Western Africa	Cross-sectional	Pregnancy termination, neonatal death, hydrocephaly, mental retardation, and chorioretinitis in infants.	Literacy level, gardening, blood transfusion, consumption of uncooked meat, and cat ownership.
Olusi et al. [25]	Nigeria	Western Africa	Cross-sectional	Lymphadenopathy and flu-like illness.	Marital status, tasting raw meat, and the presence of rodents or cockroaches.
Murebwayire et al. [26]	Rwanda	Eastern Africa	Cross-sectional	Spontaneous abortions, congenital deformities.	Drinking untreated water and ingestion of undercooked meat.
Ndiaye et al. 2019 [27]	Senegal	Western Africa	Cross-sectional	Severe pregnancy complication.	Consumption of raw or undercooked meat and contaminated food or water.
Yusuf et al. [28]	Somalia	Eastern Africa	Cross-sectional	Abortion and congenital defects.	Contact with cats and consumption of raw or undercooked meat.
Mustafa et al. [29]	Sudan	Eastern Africa	Cross-sectional	Severe foetal consequences in congenital transmission.	Contact with cats, ingestion of raw meat.
Lushina et al. [5]	Tanzania	Eastern Africa	Cross-sectional	Asymptomatic miscarriage, swollen glands(lymphadenopathy), diarrhoea, Leg swelling, nausea, blindness, eclampsia, Anaemia, and gestational diabetes.	Maternal age, consumption of undercooked meat, and poor hygiene.
Paul et al. [6]	Tanzania	Eastern Africa	Cross-sectional	Asymptomatic, congenital anomalies.	Consumption of undercooked meat, raw vegetables, soil contact, and drinking contaminated water.
Rukamba et al. [3]	Uganda	Eastern Africa	Cross-sectional	Intrauterine growth retardation, mental retardation, miscarriages, and congenital infections.	Contact with cats, drinking untreated water, and HIV status.
Daka et al. [30]	Zambia	Southern Africa	Cross-sectional	Miscarriage, stillbirth.	Non-significant determinants.
Frimpong et al. [31]	Zambia	Southern Africa	Cross-sectional	Ocular disease, lymphadenopathy, encephalitis.	Contact with cats.

## Data Availability

Since this study is based on published literature, no new primary datasets were created. The manuscript and its Supplementary Materials contain the extracted and analysed data that support the review’s conclusions. The Open Science Framework (OSF) was used to prospectively register this scoping review. In line with the registered protocol and to ensure transparency and reproducibility, the following materials have been made publicly available in the OSF repository: (i) the complete data extraction form, (ii) the study codebook, (iii) Studies with justification, (iv) search strategy with search string and deduplication process. Access to these resources is available at Open Science Framework (OSF) with registration number 10.17605/OSF.IO/PDZBC. On reasonable request, the corresponding author may provide more information about the extracted data.

## Supplementary Materials

The following supporting information can be downloaded at: https://www.mdpi.com/article/10.3390/tropicalmed11040104/s1, Table S1: PRISMA_2020_checklist; Supplementary File S1 provides the complete search strategies for each database (PubMed, Scopus, Google Scholar, and ResearchGate), including search dates, full search strings, and applied filters. As well as the data-base breakdown and deduplication summary, detailing the number of records retrieved per data-base, the number of duplicates removed, and the final counts after deduplication. Supplementary File S1 contains the data extraction form, the accompanying codebook, and a complete list of full-text studies excluded, along with their justifications.

## Abbreviations

ELISA	Enzyme-Linked Immunosorbent Assay
IgG	Immunoglobulin G
IgM	Immunoglobulin M
MAT	Modified Agglutination Test
PCR	Polymerase Chain Reaction
RDT	Rapid diagnostic test
EIA	Enzyme Immunoassay
*T. gondii*	*Toxoplasma gondii*
OSF	Open Science Framework
