# Supplementary material for: Early Warning Signs, Effects, Risk Factors, and Diagnostic Indicators of Toxoplasmosis in Pregnant Women in Africa: A Scoping Review
Source: Trop Med Infect Dis. 2026 Apr 17;11(4):104. doi: 10.3390/tropicalmed11040104 (PMC13120289; doi:10.3390/tropicalmed11040104)
Supplement: Supplementary file 1 [file tropicalmed-11-00104-s001.zip › Supplementary File S1.pdf]

Complete Search Strategies and Deduplication Process

Systematic Review Title: Early Maternal Warning Signs, Pregnancy and Fetal Outcomes, Risk Factors and Diagnostic Approaches of Toxoplasma gondii Infection in Pregnant Women (2000–2025)

Complete Search Strategies and Deduplication Process

1. Search Dates; Initial search conducted: 07 October 2025; Final updated search conducted: 08 December 2025.

Time restriction: January 2000 – December 2025

Language restriction: English

2 PubMed Search Strategy

Database- PubMed

Interface- National Library of Medicine (NLM)

Last Search Date- 08 December 2025

Years Covered: January 2000 – December 2025

("Search: (Full Search String ("Toxoplasmosis, Congenital"[MeSH] OR "Toxoplasma"[MeSH] OR "Toxoplasmosis"[MeSH] OR "Toxoplasma gondii"[tiab] OR "T. gondii"[tiab]) AND ("Pregnancy"[MeSH] OR "Pregnant Women"[MeSH] OR "Pregnancy Complications, Infectious"[MeSH] OR "Fetus"[MeSH] OR "prenatal"[tiab] OR "fetal"[tiab] OR "foetal"[tiab] OR "gestation"[tiab]) AND ("Early Diagnosis"[MeSH] OR "Symptom Assessment"[MeSH] OR "Risk Factors"[MeSH] OR "early warning"[tiab] OR "signs"[tiab] OR "symptoms"[tiab] OR "clinical features"[tiab] OR "risk factor"[tiab] OR "transmission"[tiab] OR "diagnostic"[tiab] OR "screening"[tiab])) AND (Full Search String ("Toxoplasmosis, Congenital"[MeSH] OR "Toxoplasma"[MeSH] OR "Toxoplasmosis"[MeSH] OR "Toxoplasma gondii"[tiab] OR "T. gondii"[tiab]) AND ("Pregnancy"[MeSH] OR "Pregnant Women"[MeSH] OR "Pregnancy Complications, Infectious"[MeSH] OR "Fetus"[MeSH] OR "prenatal"[tiab] OR "fetal"[tiab] OR "foetal"[tiab] OR "gestation"[tiab]) AND ("Early Diagnosis"[MeSH] OR "Symptom Assessment"[MeSH] OR "Risk Factors"[MeSH] OR "early warning"[tiab] OR "signs"[tiab] OR "symptoms"[tiab] OR "clinical features"[tiab] OR "risk factor"[tiab] OR "transmission"[tiab] OR "diagnostic"[tiab] OR "screening"[tiab])) Filters: Free full text, from 2000/1/1 - 2025/12/1

Filters applied: Language: English, Publication date: 01 January 2000 – 31 December 2025

3. Database 2: Scopus

Database-Scopus

Interface- Elsevier

Scopus Search Strategy

Last Search Date- 08 December 2025

Years Covered: January 2000 – December 2025

Full Search String- (TITLE-ABS-KEY("Toxoplasma gondii") OR TITLE-ABS-KEY("T. gondii") OR TITLE-ABS-KEY("toxoplasmosis")) AND (TITLE-ABS-KEY("pregnant") OR TITLE-ABS-KEY("fetal") OR TITLE-ABS-KEY("foetal") OR TITLE-ABS-KEY("prenatal") OR TITLE-ABS-KEY("gestation")) AND (TITLE-ABS-KEY("early warning") OR TITLE-ABS-KEY("signs") OR TITLE-ABS-KEY("symptoms") OR TITLE-ABS-KEY("risk factors") OR TITLE-ABS-KEY("clinical features") OR TITLE-ABS-KEY("diagnostics"))

4. Google Scholar Search Strategy

Search conducted on 08 December 2025.

Search terms used:

"Toxoplasma gondii" AND "pregnant women" AND ("clinical signs" OR symptoms OR "risk factors" OR diagnosis)

Results sorted by relevance

First 200 records screened

Inclusion restricted to English-language publications (January 2000–December 2025)

5. ResearchGate Search

ResearchGate Search

ResearchGate was used as a supplementary source to identify grey literature and retrieve full-text articles not accessible through indexed databases. It was not considered a primary bibliographic database.

Search terms applied:

"Toxoplasma gondii" AND "pregnant women" AND (symptoms OR "clinical signs" OR diagnosis)

All records retrieved were:

Exported manually into Zotero

Checked for duplication against indexed database records

Screened using the same predefined inclusion and exclusion criteria

Subjected to identical data extraction and quality assessment procedures

Your revised search strategy now satisfies PRISMA 2020 requirements:

Database Yield and Deduplication Summary

Database	Records Retrieved	Duplicates Removed	Records After Deduplication
PubMed	108	54	54
Scopus	93	57	36
Google Scholar (first 200 screened)	137	77	60
ResearchGate	69	47	22
**Total**	**407**	**235**	**172**

Screening and Eligibility

Screening Stage	Number
Records excluded after title/abstract screening	80
Full-text articles assessed	92
Full-text articles excluded	61
Studies included in the final review	31

Reasons for Full-Text Exclusion (n = 61)

- Reported only seroprevalence data without clinical, maternal, or fetal correlates;
- Did not present primary data (e.g., reviews, meta-analyses, editorials, commentaries);
- Focused exclusively on animal studies without pregnant women;
- Were case reports or small case series lacking adequate methodological detail
- Lacked essential bibliographic or methodological information;
- Were inaccessible after reasonable attempts to obtain the full text.
- Studies reporting exclusively fetal outcomes without maternal clinical data were included only if they contributed to the secondary objective of characterising pregnancy and congenital outcomes.

The complete search strategies for each database, including full search strings, search dates, language restrictions, and study-type filters, are provided in this supplementary file. Records retrieved from the database and manual searches were imported into Zotero for management and deduplication. Duplicate removal was performed using Zotero's automated detection tools, followed by manual verification. Because a portion of the search process involved manual web-based identification and direct article downloads before database classification, database source attribution was retrospectively assigned where possible. The full Zotero reference library used for screening and deduplication can be made available upon reasonable request to support transparency and reproducibility.

DATA EXTRACTION FORM

Systematic Review Title: Early Maternal Warning Signs, Pregnancy and Fetal Outcomes, Risk Factors and Diagnostic Approaches of Toxoplasma gondii Infection in Pregnant Women (2000–2025)

SECTION A: Study Identification

Variable	Data Entry
Study ID (Reviewer Assigned)	
First Author	
Year of Publication	
Full Citation	
Country	
Geographical Region	Northern / Western / Central / Eastern / Southern Africa / Europe / Middle East / Latin America
Journal	
DOI	

SECTION B: Study Characteristics

Variable	Data Entry
Study Design	Cross-sectional / Cohort / Case–control / Surveillance / Longitudinal
Study Setting	Antenatal clinic / Hospital-based / Community-based / Surveillance
Sample Size	
Study Population	Pregnant women (trimester if reported)
Study Period	

SECTION C: Maternal Early Clinical Signs

(Extract only if explicitly reported)

Variable	Yes/No	Details
Maternal symptoms reported	☐ Yes ☐ No	
Lymphadenopathy / Swollen glands		
Fever / Febrile illness		
Malaise		
Flu-like illness		
Nausea		
Mild non-specific symptoms		
Acute infection (unspecified)		
Asymptomatic infection		
Other		

SECTION D: Pregnancy and Fetal Outcomes

(Grouped according to revised manuscript structure)

Variable	Yes/No	Details
Miscarriage / Spontaneous abortion		
Congenital anomalies (malformation, hydrocephalus, cerebral calcification, neurological impairment)		
Neonatal / Fetal loss (stillbirth, neonatal death, fetal loss)		
Ocular complications		
Pregnancy complications (incl. IUGR)		
Congenital infection (unspecified)		
Labour/delivery complications		
Other		

SECTION E: Risk Factors

1. Dietary Factors

Variable	Yes/No	Details
Undercooked/raw meat		
Raw vegetables/fruits		
Unpasteurised milk		
Unsafe/untreated drinking water		
Other dietary exposure		

2. Environmental Factors

Variable	Yes/No	Details
Cat ownership/contact		
Cat litter exposure		
Soil contact/gardening		
Contaminated water exposure		
Rodents/cockroaches		
Poor hygiene practices		
Other		

3. Socioeconomic Factors

Variable	Yes/No	Details
Education level		
Residence (urban/rural)		
Occupation		
Population density		
Marital status		
Awareness/knowledge level		
Income		

4. Obstetric / Host Factors

Variable	Yes/No	Details
Maternal age		
Parity		
Previous spontaneous abortion		
HIV status		
Blood transfusion history		
Immune/genetic factors		
Trimester		
Other		

SECTION F: Diagnostic Methods and Indicators

Diagnostic Category (Select One Primary Category)

☐ Enzyme-based immunoassay (ELISA, EIA, EUROIMMUN, IgG/IgM, IgG avidity)
☐ Agglutination-based (LAT, DAT)
☐ Molecular (PCR)
☐ Combined serology + molecular (ELISA + PCR)
☐ Rapid diagnostic test (RDT)

Specific Diagnostic Details

Variable	Data Entry
Specific Test Name	
IgG measured	Yes/No
IgM measured	Yes/No

IgG avidity performed	Yes/No
PCR target (if applicable)	
Seroprevalence (%)	
Acute infection prevalence (%)	
Congenital infection detection	

SECTION G: Notes for Reviewers

Variable	Data Entry
Key Findings Relevant to Early Warning Framework	
Any Data Ambiguities	
Comments	

Supplementary File S1: Data Extraction Codebook

Systematic Review Title: Early Warning Signs, Pregnancy Outcomes, Risk Factors and Diagnostic Approaches of Toxoplasma gondii Infection Among Pregnant Women

1. General Study Identification Variables

Variable Name	Description	Coding Format	Example
Study_ID	Unique identifier assigned to each included study	Numeric (1–31)	1
First_Author	First author surname	Text	Adeniyi
Year	Year of publication	YYYY	2023
Country	The country where study was conducted	Text	Nigeria
Region	geographical classification	AFRO / EMRO / EURO / PAHO	AFRO
Study_Design	Type of study design	1=Cross-sectional; 2=Case-control; 3=Cohort; 4=Longitudinal; 5=Retrospective; 6=Surveillance	1
Study_Setting	Health facility/community/tertiary hospital	Text	Antenatal clinic
Study population	Total number of pregnant women included	Numeric	350

2. Maternal Early Warning Signs

(Record only if explicitly reported)

Variable Name	Description	Coding
Any_Maternal_Signs	Whether maternal clinical signs were reported	0=No; 1=Yes
Fever	Reported fever	0=No; 1=Yes

Lymphadenopathy	Swollen lymph nodes	0=No; 1=Yes
Headache	Reported headache	0=No; 1=Yes
Myalgia	Muscle pain	0=No; 1=Yes
Flu_like_Symptoms	Flu-like syndrome	0=No; 1=Yes
Visual_Disturbance	Ocular involvement	0=No; 1=Yes
Asymptomatic	No clinical symptoms reported	0=No; 1=Yes
Other_Maternal_Signs	Specify if reported	Text

3. Fetal / Neonatal Outcomes

(Combined categories as per revised classification)

Variable Name	Description	Coding
Any_Fetal_Outcome	Whether fetal outcomes were assessed	0=No; 1=Yes
Congenital_Anomalies	Includes hydrocephalus, cerebral calcification, neurological impairment, congenital malformation/defect	0=No; 1=Yes
Neonatal_Foetal_Loss	Includes stillbirth, neonatal death, foetal loss	0=No; 1=Yes
Pregnancy_Complication	Includes severe pregnancy complications & intrauterine growth restriction	0=No; 1=Yes
Preterm_Birth	Preterm delivery	0=No; 1=Yes
Low_Birth_Weight	LBW reported	0=No; 1=Yes
Other_Fetal_Outcomes	Specify	Text

4. Risk Factors

4.1 Dietary Factors

Variable	Coding
Raw_Undercooked_Meat	0=No; 1=Yes
Unwashed_Vegetables	0=No; 1=Yes
Unpasteurized_Milk	0=No; 1=Yes
Untreated_Water	0=No; 1=Yes
Street_Food	0=No; 1=Yes

4.2 Environmental Factors

Variable	Coding
Cat_Ownership	0=No; 1=Yes
Cat_Contact	0=No; 1=Yes
Soil_Contact	0=No; 1=Yes
Farming_Activities	0=No; 1=Yes
Poor_Sanitation	0=No; 1=Yes

4.3 Socioeconomic Factors

Variable	Coding
Low_Education	0=No; 1=Yes
Rural_Residence	0=No; 1=Yes
Low_Income	0=No; 1=Yes

Occupation_Risk	0=No; 1=Yes

4.4 Obstetric / Host Factors

Variable	Coding
Gravidity	Numeric
Trimester	1=First; 2=Second; 3=Third
History_Miscarriage	0=No; 1=Yes
HIV_Status	0=Negative; 1=Positive; 9=Not reported
Immunocompromised	0=No; 1=Yes

5. Diagnostic Methods

Variable Name	Description	Coding
Diagnostic_Category	1=Serological; 2=Molecular; 3=Mixed	
IgG_Test	0=No; 1=Yes	
IgM_Test	0=No; 1=Yes	
IgG_Avidity	0=No; 1=Yes	
ELISA	0=No; 1=Yes	
Rapid_Test	0=No; 1=Yes	
PCR	0=No; 1=Yes	
Digital_PCR	0=No; 1=Yes	
Direct_Agglutination	0=No; 1=Yes	

6. Seroprevalence Data

Variable	Format
IgG_Prevalence	Percentage (%)
IgM_Prevalence	Percentage (%)
Overall_Seroprevalence	Percentage (%)
Acute_Infection	0=No; 1=Yes
Chronic_Infection	0=No; 1=Yes

7. Quality Assessment Variables (if applicable)

Variable	Coding
Risk_of_Bias_Score	Numeric
Risk_of_Bias_Level	1=Low; 2=Moderate; 3=High

Coding Notes

“NR” = Not Reported

Binary variables coded as 0/1

Percentages recorded as presented in the original studies

Multiple responses allowed for risk factors

Combined fetal outcome categories follow the revised classification approved during manuscript revision

List of Excluded Studies

Systematic Review Title: Early Warning Signs, Pregnancy Outcomes, Risk Factors and Diagnostic Approaches of Toxoplasma gondii Infection Among Pregnant Women

1.	(Yosef et al.	2024)
2.	(Polanunu et al.	2021)

3.	(Mohaghegh et al.	2016)
4.	(Kurere et al.	2024)
5.	(Abdelbaset et al.	2020)
6.	(Alqaisi et al.	2021)
7.	(Aksoy Sanay et al.	2024)
8.	(Lima et al.	2024)
9.	(Deganich et al.	2022)
10.	(Damar Çakırca et al.	2023)
11.	(Yohanes et al.	2017)
12.	(Khammari et al.	2014)
13.	(Barzgar et al.	2024)
14.	(Sroka et al.	2010)
15.	(Laguardia et al.	2024)
16.	(Evangelista et al.	2020)
17.	(Vueba et al.	2020)
18.	(Tarekegn et al.	2020)
19.	(Mulu Gelaw et al.	2024)
20.	(Rabaan et al.	2023)
21.	(Mwambe et al.	2013)
22.	(İnceboz et al.,)	(2021)
23.	(Amagbégnon et al.	2023)
24.	(Ullah et al.	2022)
25.	(Garee et al.	2022)
26.	(Akubuilo et al.	2020)
27.	(Pandey	2018)
28.	(Briciu et al.	2023)
29.	(Okojokwu et al.	2022)
30.	(Sadeghi Dehkordi et al.	2022)
31.	(Njunda et al.	2011)
32.	(Hajra Mateen	2024)
33.	(Ayeah et al.	2022)
34.	(Moshfeghi et al.	2017)
35.	(Bartholo et al.	2020)
36.	(Rehman et al.	2020)
37.	(Das et al.	2016)
38.	(Bieńkowski et al.	2022)
39.	(Varella et al.	2003)
40.	(Teimouri et al.	2024)
41.	(Khademi et al.	2019)
42.	(Mousavi et al.	2018)
43.	(Saad et al.	2020)
44.	(Negero et al.	2017)
45.	(Singh et al.	2021)
46.	(Adugna et al.	2021)
47.	(Yasodhara et al.	2004)
48.	(Singh	2003)
49.	(Hassanen et al.	2023)
50.	(Nowakowska et al.	2005)
51.	(Csep et al.	2021)
52.	(Paul et al.	2022)

53.	(Tűzkő et al.	2024)
54.	(Teweldemedhin et al.	2019)
55.	(Fenta	2019)
56.	(Bashour et al.	2024)
57.	(Bamba et al.	2017)
58.	(Al-Adhroey et al.	2019)
59.	(Abamecha & Awel	2016)
60.	(Raso et al.	2025)
61.	(Di Carlo et al.	2011)

Full list of 61 studies excluded after full-text eligibility assessment

Full-text articles were excluded because they:

- Reported only seroprevalence data without clinical, maternal, or fetal correlates;
- Did not present primary data (e.g., reviews, meta-analyses, editorials, commentaries);
- Focused exclusively on animal studies without pregnant women;
- Were case reports or small case series lacking adequate methodological detail
- Lacked essential bibliographic or methodological information;
- Were inaccessible after reasonable attempts to obtain the full text.
- Studies reporting exclusively fetal outcomes without maternal clinical data were included only if they contributed to the secondary objective of characterising pregnancy and congenital outcomes.
